# Disaggregating Middle Eastern and North African Populations in Mortality Records to Better Understand COVID-19 Inequalities in Virginia

**DOI:** 10.1007/s40615-025-02589-1

**Published:** 2025-08-15

**Authors:** Elyas Bakhtiari

**Affiliations:** Department of Sociology, William and Mary, Williamsburg, VA USA

**Keywords:** Covid-19, Middle Eastern and North African, Health inequalities, Machine learning

## Abstract

Many ethno-racial minority populations had disproportionately high rates of COVID-19 infection and mortality during the initial years of the pandemic, but it is currently unclear whether Middle Eastern and North African populations in the USA experienced similar inequalities. This study disaggregates the MENA population in Virginia death records using a long short-term memory machine learning model that can probabilistically identify ethnically-unique names. The results suggest that MENA populations had higher overall excess mortality and a higher percentage of COVID-19 deaths among total deaths, when compared to the non-Hispanic White population of Virginia. The paper highlights the importance of disaggregating the MENA population in health inequalities research and offers a new method that improves upon previous name-based disaggregation methods.

**Supplementary Information:**

The online version contains supplementary material available at https://doi.org/10.1007/s40615-025-02589-1.

## Introduction

Many ethno-racial minority populations in the USA have been disproportionately affected by the COVID-19 pandemic, with higher rates of COVID-19 infection, hospitalization, and mortality when compared to non-Hispanic White Americans [1–4]. This has not only been observed for populations with longstanding disparities in overall mortality, such as African American and Native American populations, but also among a number of immigrant populations, which prior to the pandemic exhibited mortality advantages relative to non-Hispanic White Americans [3, 5–7]. However, tracking data used to generate such comparisons typically do not include foreign-born and US-born individuals with ancestry from Southwest Asia and North Africa, commonly referred to as the Middle East and North Africa (MENA). As a result, it is largely unclear whether MENA populations experienced similar adverse outcomes as other ethno-racial minority or immigrant populations during the COVID-19 pandemic.

The lack of identifying information for MENA populations is related to how ethnic and racial categories have been institutionalized in the USA. Many sources of health information rely on a standard established by the US Office of Management and Budget in 1978, which formally defined the white category on the US Census to include individuals with ancestry from the Middle East and North Africa, in addition to Europe [8]. However, social science research has recently suggested that the everyday experiences of ethno-racial identity and discrimination do not match this formal classification for many MENA Americans, who may not perceive themselves to be White or may not be perceived as White by others [9–13]. Importantly, racialized experiences of individual and institutionalized discrimination may contribute to health risks that are not regularly monitored due to the lack of identifying information [14–18]. In addition, ethnic identity distinctions for both Arab and non-Arab MENA populations may also shape a variety of outcomes related to health in general and the COVID-19 pandemic in particular [19, 20].

Initial evidence suggests further research into MENA American COVID-19 outcomes is warranted. An analysis of the Michigan Disease Surveillance System found that Arab Americans in Michigan had 2.6 times the odds of testing positive for COVID-19 in the first year of the pandemic when compared to non-Hispanic Whites [21]. A smaller-scale study in El Cajon, California, found similar high rates of positivity but lower odds of hospitalization and in-hospital mortality [22]. In Toronto, infection and hospitalization rates for Middle Eastern populations were 3–4 times that of the White population [23]. A number of studies have documented barriers to vaccine uptake and access to health information, although they have not directly linked them to outcomes [24–27]. To date, most studies have focused on Arab members of the MENA population, and no published studies have examined the COVID-19 mortality outcomes of MENA populations in the USA.

The lack of identifying information for MENA populations in health and mortality records presents a barrier for such research. The US Office of Management and Budget has announced plans to add a “Middle Eastern and North African” category to the 2030 census to improve data availability about MENA populations, but in the meantime, researchers have had to rely on other sources of information to address gaps in data availability [28]. Some prior studies have used name-matching techniques that probabilistically identify likely MENA records based on given name and surname similarity to ethnically- or regionally-distinct name lists [29–32]. Naming patterns have long been studied as markers of ethnic identity and immigrant integration patterns [33–35]. This study relies on a similar probabilistic classification approach but develops a machine-learning method that addresses two limitations of the direct-matching method. First, the machine-learning model does not require an external list of names and can be trained on a foreign-born subset within the data. Second, the machine-learning approach can assign probabilities for names without direct matches to an external list, as is the case with unique transliterations or uncommon names.

For this study, a long short-term memory recurrent neural network was trained on names from a subset of foreign-born individuals in health records from the Virginia Department of Health, and the trained model was then applied to the full set of mortality records from 2015 to 2022 for the state of Virginia, which has the third largest MENA population in the USA by relative size [36].^1^ The study seeks to disaggregate the MENA population from conventional identifiers of ethnicity and race in mortality records in order to test for relative inequalities in COVID-19 mortality for MENA Virginians, particularly in comparison to the non-Hispanic White population of the state.

## Materials and Methods

Mortality data for this study come from the vital statistics records for the state of Virginia provided by the Virginia Department of Health. Data span 2015–2022 and include ICD-10 code U07.1 for cause of death attributed to COVID-19. The data also include each decedent’s given name, surname, race, ethnicity, and place of birth, which are used to identify the MENA population.

Country of birth information in the records was used to identify MENA immigrants. Records with a country of birth from the following countries were classified in the MENA group, based on FIPS codes used to classify origin countries: Afghanistan, Algeria, Bahrain, Egypt, Iran, Iraq, Jordan, Kuwait, Lebanon, Libya, Morocco, Oman, Palestine, Qatar, Saudi Arabia, Syria, Tunisia, United Arab Emirates, and Yemen.^2^

Because parental country of birth information was not available, a name-classification algorithm was used to probabilistically identify US-born descendants of MENA immigrants based on similarity in name characteristics. First, the given name and last name of each record were encoded using byte pair encoding, a method for breaking names into subclusters of letter combinations, and a long short-term memory recurrent neural network was trained on the encoded names in order to distinguish both character clusters and sequences unique to MENA names. The model was trained on a foreign-born subset of 799,532 VDH birth and mortality records, with an out-of-sample test precision of 0.82, a recall score of 0.83, and an overall F1 score of 0.82. The trained model was then used to predict MENA background for names in the full set of death records. For respondents that were not identified as MENA by the algorithm, race and ethnicity variables were used to code the following categories: non-Hispanic White, non-Hispanic Black, Hispanic, Asian and Pacific Islander American, and Other. Additional details about the name-classification approach, including its performance relative to conventional direct-matching methods, can be found in the accompanying appendix.^3^

Because population denominators can be challenging to calculate for such a small and hard-to-define population, particularly given documented data collection challenges during 2020, two measures that can be calculated solely using mortality data were used to assess outcomes during the COVID-19 pandemic: Excess all-cause mortality and percentage of deaths due to COVID-19. Excess deaths were measured as the ratio of observed to expected deaths for 2020–2022, using a baseline of predicted deaths calculated from trends in monthly deaths for each ethno-racial group from 2015 to 2019. Predicted deaths were calculated using Poisson regression models that adjust for temporal trends and seasonal components in the death series data from 2015 to 2019. These models were then used to generate monthly death predictions for each ethno-racial group during the pandemic years. For a subset of analyses that distinguished populations by nativity, annual trends were used to calculate an expected death baseline due to the smaller sample sizes.

The second measure, proportion of deaths due to COVID-19, was calculated as the number of deaths with U07.1 listed as an attributed cause of death divided by the total number of deaths for each ethno-racial, sex, nativity, and year combination. Although there has been documented underreporting of COVID-19 deaths in some periods and localities in the USA, research suggests excess deaths that were not attributed to COVID-19 may represent unrecognized COVID-19 mortality [37].

## Results

Table 1 contains the descriptive statistics for each group. There are notable sociodemographic characteristics of the MENA population in Virginia that may affect its mortality patterns during the pandemic. Approximately 56% of all MENA individuals in the VDH death records were classified as foreign-born. Although immigrant populations have been found to have lower mortality in many circumstances, emerging evidence suggests this might not have been the case during the COVID-19 pandemic [3, 5–7]. Other characteristics might also affect the mortality profile of MENA Virginians. The population is younger than the non-Hispanic White population on average (measured both as age-of-death in the death records and in population estimates from the ACS) and had one of the highest levels of education among groups in the sample.

**Table 1:** Descriptive statistics for Virginia death records, 2015–2022

	APIA	Hispanic	MENA	Non-Hispanic Black	Non-Hispanic White	Other
Proportion foreign born	0.84	0.61	0.56	0.03	0.03	0.29
Proportion female	0.49	0.45	0.52	0.48	0.49	0.44
Age at death (mean)	72.73	60.35	71.29	68.51	75.3	64.92
Education (mean)	4.3	3.16	4.36	3.2	3.67	3.59
COVID-19 deaths	648	1069	209	4836	14,966	55
Total deaths						
2015–2019 (mean)	1528	1312	604	13,415	50,725	189
2020	2115	2234	739	17,079	57,924	206
2021	2212	2265	786	17,973	61,935	247
2022	2130	2099	775	16,521	57,115	325

Data come from the Virginia Department of Health vital statistics records, 2015–2022

The descriptive count of mortality reveals the MENA population in Virginia averaged 604 deaths per year between 2015 and 2019, and that jumped to 739 in 2020 and 786 in 2021, before dropping slightly to 775 in 2022. This represented an increase of 22.4%, 30.1%, and 28.3% above pre-COVID trends, respectively. This was a larger increase than observed among non-Hispanic White Virginians, whose deaths increased by 14.2% in 2020, 22.1% in 2021, and 12.6% in 2022, relative to pre-pandemic trends. The proportional increase in deaths for MENA Virginians was comparable to the relative increase for non-Hispanic Black Virginians (with increases of 27.3%, 34.0%, and 23.2%) but lower than the relative increases seen among APIA and Hispanic populations.

**Fig. 1:**
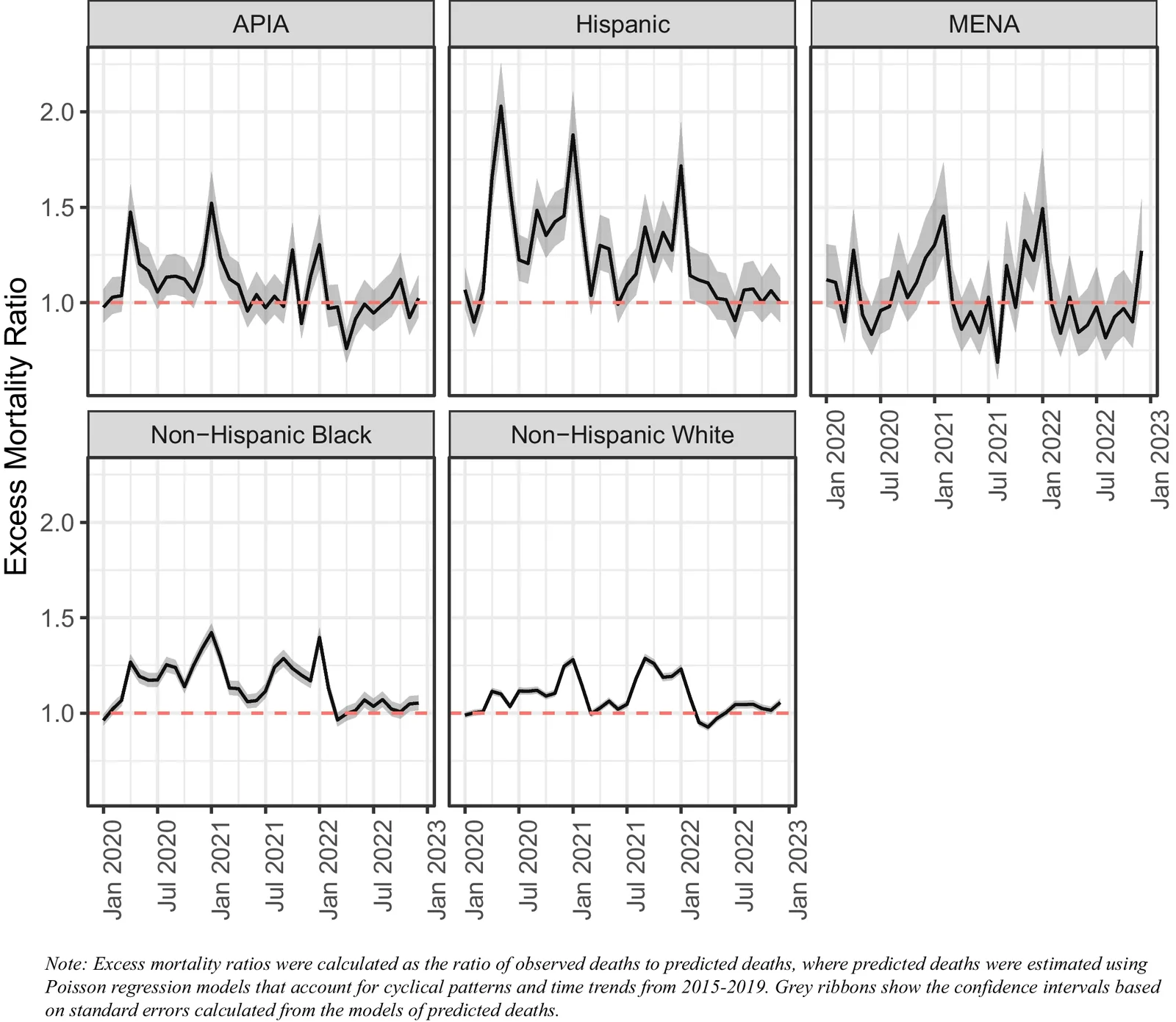
Excess mortality ratio by ethno-racial group in Virginia, 2020–2022

This relative change in mortality is more formally modeled in Fig. 1, which presents the estimates of excess mortality for each ethno-racial category. These estimates account for linear trends and cyclical patterns in deaths to establish baseline predictions for expected deaths during these years. Like other populations, the excess mortality ratio—the observed deaths divided by the expected deaths predicted by the Poisson regression model—peaked first in April 2020 (1.28, CI 1.12–1.49) during the initial wave of the COVID-19 pandemic, again in winter of 2021 (1.45, CI 1.25–1.73) during a second wave, and in January 2022 (1.49, CI: 1.27–1.81) during the “Omicron wave.” The MENA excess mortality peaks were notably higher than those for the non-Hispanic White population, which had an initial excess mortality peak of 1.11 (CI 1.10–1.13) in April 2020, a winter peak of 1.28 (CI 1.26–1.30) in January 2021, and an Omicron-wave high of 1.23 (CI 1.21–1.25) in January of 2022. Compared to other ethno-racial minority populations, the MENA excess mortality peaks were comparable to APIA and non-Hispanic Black patterns in magnitude, though substantially smaller than the patterns for the Hispanic population. Whereas some groups had lower excess mortality ratios by January 2022, the MENA population did not see such a decline and had the second-highest mortality ratio during that wave.

Table 2 presents estimates of excess mortality disaggregated by nativity status. Because of the small monthly number of deaths among some immigrant populations, these results establish a baseline of expected deaths based on pre-pandemic annual deaths data, rather than monthly trends. Across most ethno-racial groups, immigrant populations had higher rates of excess mortality than their US-born counterparts. Among the MENA population, the ratio of observed to expected deaths from immigrants was 1.13 (CI 1.01–1.27) in 2020, compared to 0.95 (CI 0.84–1.09) for the US-born MENA population. By 2022, the excess mortality ratio for MENA immigrants in Virginia was 1.16 (CI 0.98–1.40) and had not declined as much as other populations; however, the large confidence interval for this estimate includes the possibility of no excess deaths.

**Table 2:** Excess mortality ratios by ethno-racial group and nativity in Virginia, 2020–2022

		2020			2021			2022		
		Ratio	CI low	CI high	Ratio	CI low	CI high	Ratio	CI low	CI high
Foreign-born										
	APIA	1.11	1.05	1.17	1.05	0.98	1.13	0.95	0.87	1.04
	Hispanic	1.51	1.41	1.62	1.28	1.17	1.41	1.10	0.99	1.24
	MENA	1.13	1.01	1.27	1.20	1.05	1.39	1.16	0.99	1.40
	Non-Hispanic Black	1.21	1.09	1.36	1.04	0.91	1.22	0.84	0.72	1.02
	Non-Hispanic White	1.11	1.05	1.17	1.08	1.01	1.15	1.03	0.95	1.12
US-Born										
	APIA	1.12	0.95	1.34	1.08	0.89	1.38	0.85	0.67	1.17
	Hispanic	1.14	1.05	1.25	1.28	1.15	1.44	1.10	0.96	1.27
	MENA	0.95	0.85	1.09	0.92	0.79	1.10	0.82	0.68	1.02
	Non-Hispanic Black	1.17	1.15	1.19	1.20	1.17	1.23	1.08	1.05	1.11
	Non-Hispanic White	1.09	1.08	1.10	1.14	1.13	1.16	1.04	1.02	1.05

Note: Excess mortality ratios were calculated as the ratio of observed deaths to predicted deaths, where predicted deaths were estimated using annual time trends from 2015 to 2019. Data come from the Virginia Department of Health vital statistics records, 2015–2022

Table 3 presents the percentage of all deaths that were attributed to COVID-19 in 2020, 2021, and 2022 for each group. Because this estimate does not rely on population denominator estimates, it can be presented at a more granular level, broken down by both sex and nativity status. In 2020, the percentage of deaths attributed to COVID-19 for MENA women was lower than percentages for non-Hispanic White Americans, whereas the percentage for foreign-born MENA men (12.6% vs. 7.9%) was substantially higher relative to their non-Hispanic White counterparts, while for US-born MENA men, it was lower. By 2022, MENA men had the highest percentage of deaths attributed to COVID-19 among foreign-born men, and foreign-born MENA women had the second-highest percentage.

**Table 3:** Percentage of deaths attributed to COVID-19 by sex, nativity, and ethno-racial group, 2020–2022

		2020		2021		2022	
		Foreign-born	US-born	Foreign-born	US-born	Foreign-born	US-born
Female							
	APIA	9.4	3.5	11.2	5.5	6.7	4.5
	Hispanic	24.7	8.2	18.6	9.2	9	7.8
	MENA	8.1	7	8.7	7.7	8.7	6
	Non-Hispanic Black	12.9	9.1	11.2	13.6	5.9	6.9
	Non-Hispanic White	7	6.9	8.2	10.6	6.4	7
Male							
	APIA	14.5	5.4	13.5	12.1	6.9	11.1
	Hispanic	32.9	6.1	26.1	10.1	10.2	5.5
	MENA	12.6	3.8	13.1	9.3	10.6	6.8
	Non-Hispanic Black	18.7	8	15.6	11.3	4.7	6.5
	Non-Hispanic White	7.9	6.7	10.2	11.4	6.2	7.9

Deaths attributed to COVID-19 were identified using the U07.1 ICD-10 code in Virginia Department of Health vital statistics records

## Discussion

This paper used a combination of country-of-birth data and a machine-learning name classification algorithm to disaggregate the COVID-19 mortality outcomes of Middle Eastern and North African populations in Virginia. The evidence suggests that, much like other ethno-racial populations, the MENA population of Virginia was disproportionately affected by COVID-19 at certain points in the pandemic when compared to the non-Hispanic White majority. The MENA immigrant population, in particular, experienced excess mortality throughout the pandemic that warrants follow-up in future research.

Estimates provided in this paper come with limitations, given the small size of the MENA population and the methods used for identifying those born in the USA. Although name-matching has become a common approach to overcoming the lack of MENA identifying information in health records, there is the possibility of misclassification due to divergent naming conventions among the second generation, overlap in naming conventions with non-MENA populations, naming conventions among inter-ethnic or inter-racial marriages not captured in the data, and errors related to the modeling strategy.

The small size of the MENA population presents its own challenges. Some of the excess mortality estimates were based on modeled predictions of monthly deaths, which fluctuated from 30 to 70 monthly deaths for the MENA population of Virginia prior to the pandemic. Cyclical trends were less evident for MENA Virginians than in other populations, due to the month-to-month noise among the smaller pool of data, and excess mortality estimates may be more sensitive to modeling approaches. Because of the small sample size, it was not feasible to further disaggregate the MENA population. However, prior scholarship has noted heterogeneity in MENA experiences and outcomes by country of origin, religion, and racial identification [12, 38, 39]. Future research on MENA populations would benefit from examining the intersection of ethnic, national, racial, and religious identities.

This study presents descriptive findings and does not attempt to offer causal explanations for MENA COVID-19 outcomes. However, the spatial distribution of the MENA population of Virginia may offer important context for understanding its COVID-19 risk. According to American Community Survey data, approximately 95% of Virginia’s MENA population lives in a metropolitan area, with the majority located in the Northern Virginia portion of the Washington D.C. metropolitan area. This may have disproportionately exposed MENA populations to COVID-19 risk in the early months of the pandemic, when incidence was higher in urban areas, particularly in the Northeast and Mid-Atlantic regions [40]. However, the spatial impact of the pandemic subsequently shifted to non-metropolitan areas, influenced by health care access, resistance to non-pharmaceutical interventions, and a variety of political and social factors [41–43]. The mechanisms shaping MENA excess mortality may have varied throughout the pandemic, and literature suggests researchers should further investigate discrimination experiences, health care access, vaccination patterns, and other social determinants of health.

Despite the limitations, the findings of this study contribute to our understanding of how the COVID-19 pandemic has disproportionately affected some ethno-racial minority populations. Scholars have recently argued that the relative “invisibility” of MENA Americans in institutionalized racial categories does not match their everyday experience of the US ethno-racial hierarchy nor does it reflect their potential risk for health problems and early mortality [14–18]. The evidence of mortality inequalities between MENA and non-Hispanic White populations in Virginia during portions of the COVID-19 pandemic supports this assertion. The lack of identifying information for MENA Americans may cause some health inequalities to go overlooked, leaving potentially vulnerable populations without access to public health and medical attention.

## Supplementary Information

Below is the link to the electronic supplementary material.

ESM 1 (297 KB PDF)

## Data Availability

The mortality data used in this study are restricted-use and cannot be shared publicly. Researchers may contact the Virginia Department of Health to inquire about access. Code use for analysis will be made available in a public repository after publication.

## Footnotes

1 See the accompanying Appendix for additional details about the MENA population of Virginia.

2 The concept of the “Middle East” is a social and political construction without clear geographical boundaries or criteria for inclusion. Some non-Arab countries on the periphery, such as Afghanistan, are included in some definitions and excluded in others. This paper uses a broad set of countries that in part reflect the immigrant population of Virginia from the Muslim-majority countries of Southwest Asia.

3 The code used for the name-classification algorithm will be made publicly available in an open repository upon publication.
