# Supplementary material for: Disaggregating Middle Eastern and North African Populations in Mortality Records to Better Understand COVID-19 Inequalities in Virginia
Source: J Racial Ethn Health Disparities. 2025 Aug 15;13(5):4404–11. doi: 10.1007/s40615-025-02589-1 (PMC13546347; doi:10.1007/s40615-025-02589-1)
Supplement: Supplementary file 1 [file 40615_2025_2589_MOESM1_ESM.pdf]

Appendix: Disaggregating Middle Eastern and North African Populations in Mortality Records to Better Understand Covid-19 Inequalities Virginia

Table of Contents

A. Name classification algorithm 3

Table A1. Performance Metrics for Machine Learning Name Classification Model 5

Table A2. Original Ethno-Racial Classification of MENA Subsample 5

B. Comparison to Direct-Matching Method 7

Table B1. Comparison of Direct-Matching and Machine Learning Methods for Identifying MENA Names 8

C. Estimating Excess Mortality 9

Figure C1: Observed and Predicted Mortality by Ethno-Racial Group in Virginia, 2015-2022 10

Table C1. Estimates of Excess Mortality Within the Virginia MENA Population, Disaggregated By Original Ethno-Racial Classification 11

D. Virginia's MENA Population 13

Table D1. Size of MENA Population in States with the Largest MENA Population, 2019 13

Figure D1. Population Estimates from the American Community Survey by Race and Ethnicity, 2015-2022 14

Figure D2. Relative Change in Population and Death Estimates, 2019-2022 16

Figure D3. Distribution of MENA Deaths Across Virginia Counties 17

A. Name classification algorithm

The foreign-born MENA population analyzed in this study was identified using place of birth. Because the records do not contain a variable identifying parental birthplace, ethnicity, ancestry, or similar identifiers, the descendants of MENA immigrants could only be identified using characteristics of given names and surnames. Many studies have used naming conventions as a marker of ethnic identity and immigrant integration. Immigrant populations can at times be distinguished by distinct naming patterns, and naming decisions can serve as a marker of ethnic affiliation for subsequent generations.

One common approach in the public health literature for identifying populations using names involves using a direct name-matching method to compare names in the target health records with an external list in which the relative uniqueness of a name can be established. The approach in this study follows the same principle, but relies on a machine-learning method rather than direct name-matching. This allows for classifying names based on character clusters within the names, rather than full matches, resulting in a larger and possibly more-representative sample (see below for a replication of the analysis using the direct-matching method).

A long short-term memory recurrent neural network was trained on a subset of foreign-born records with classifiable MENA background (based on country of birth), and then used to predict the MENA name similarity with U.S.-born records. The VDH also provided a dataset of birth records, which were used for name-training purposes but otherwise not analyzed in this study. The name of the mother and father within the birth records were treated as having the same birth country and added to the list of names to

create a larger training dataset. This resulted in 799,532 total training names, of which 36,155 were classified as MENA. A subset of individuals born in South Asia was also included as a separate category to help the model distinguish the two nearby regions.

The first step in the training process involves encoding the given name and surname combination using byte pair encoding. This method begins by tokenizing the names as single-character representations, and it then iteratively merges the most frequently adjacent pairs of characters to form new tokens of variable character length. This process continues until a specified vocabulary size is reached or no further pairs can be merged. For example, in Arabic or Persian names, the sequence "kh" might frequently occur together and thus would be merged into a single token early on. Gradually, BPE builds a hierarchy of tokens, ranging from individual characters to common subwords like "Abdul", which are prevalent in the dataset of names. This hierarchical tokenization allows the model to efficiently process and learn from the character patterns that are characteristic of different languages and name etymologies.

The encoded names were used to train a Long Short Term Memory (LSTM) model, which was adapted from the Python ethnicolr package.[1] Long Short-Term Memory (LSTM) networks are a specialized type of Recurrent Neural Network (RNN) capable of learning long-term dependencies. LSTMs are particularly adept at tasks involving sequences, such as language processing, because of their structure, which includes memory cells and multiple gates that gates effectively allow the network to retain or forget information over long periods. When applied to encoded names, the

[1] Sood G, Laohaprapanon S. Predicting Race and Ethnicity From the Sequence of Characters in a Name. ArXiv180502109 Stat [Internet]. 2018 [cited 2021 Mar 26]; Available from: http://arxiv.org/abs/1805.02109

LSTM can detect not only individual character patterns but also their order within the names, which is crucial for recognizing linguistic variations and multiple spellings of names with similar origins. The model was trained on token windows of 25, with a batch size of 32 and 12 epochs.

Table A1. Performance Metrics for Machine Learning Name Classification Model

Group	Precision	Recall	F1-Score	Support
APIA	0.85	0.85	0.85	18674
Black	0.79	0.68	0.73	26192
Hispanic	0.93	0.92	0.93	31344
MENA	0.83	0.82	0.83	7075
Other	0.00	0.00	0.00	2476
White	0.83	0.92	0.87	60858

The name list was divided into a training set of an 80% sample of the names. Table A1 presents evidence of the model effectiveness on the remaining 20% test data set. For MENA-classified records, precision was .82, recall was .83, and the overall F1 score was .82. Although the model was not as effective for predicting other ethno-racial categories, particularly foreign-born non-Hispanic Black names, the LSTM model was only used for MENA classification in the analysis.

Table A2. Original Ethno-Racial Classification of MENA Subsample

Original Coding	N	Percent of Total
APIA	199	4.0
Hispanic	30	0.6
Non-Hispanic Black	471	9.6
Non-Hispanic White	3947	80.2
Other	273	5.5

As seen in Table A2, nearly 80% of names classified as MENA were previously classified as non-Hispanic White, which is consistent with institutionalized classification schemes. Although the majority of MENA records were previously classified as non-

Hispanic white, MENA records represented only about 1% of the total non-Hispanic white population of death records. Because this is a small population, disaggregation does not have a large impact on mortality estimates of other groups.

B. Comparison to Direct-Matching Method

Previous research has relied on a direct name-matching method to classify MENA records. This method has been used previously to identify MENA populations by matching names in records with an external list of names with known MENA ancestry. The machine-learning method used in this study was compared with a direct-matching alternative, using a list of names provided by the Social Security Administration that includes country of origin information for foreign-born individuals who submitted a social security application. This is arguably one of the most nationally-representative and largest name lists given its source from administrative records. For each given name and surname, its occurrences among MENA immigrants are calculated relative to the total number of occurrences, providing a way to probabilistically classify name combinations. Names were included if they had at least five occurrences associated with at least one MENA-origin country. The proportional representation of each name was calculated, and the resulting source file included 40,450 given names and 45,939 surnames. Following similar studies, a positive match was identified if the combination of scores for the given name and surname was higher than .15.

Many names that were classified as MENA by the LSTM algorithm did not have a direct match in the source name list. Of the total 583,545 names in the final dataset, 6% did not have a direct match for both the first or last name, and more than 56% were missing a match for one of the names.

Table B1 compares the performance of the two approaches. Because the VDH data do not contain an identifying variable for records classified as MENA and born in the United States, a subset of foreign-born MENA records was used, with a positive

identification of someone born in the MENA considered a true match for each algorithm. In addition to resulting in fewer matches, the direct-matching method was also less accurate across metrics. The LSTM method had an overall F1 of 0.83, compared to 0.51 for the direct-matching method, suggesting it was more successful at identifying true positives and minimizing both false negative and false positive matches.

Table B1. Comparison of Direct-Matching and Machine Learning Methods for Identifying MENA Names

	Direct Matching	LSTM Machine Learning
Precision	0.48	0.83
Recall	0.51	0.82
F1-Score	0.51	0.83

C. Estimating Excess Mortality

Excess mortality was estimated as the number of observed deaths once the Covid-19 pandemic began, relative to the number of deaths that would have been predicted had the pandemic not occurred, based on 2015-2019 trends. In order to establish a baseline of predicted deaths, a poisson regression model was used to account for both temporal trends and cyclical patterns:

$$D_m = \exp(\beta_0 + \beta_1 T + \sum_{i=1}^{11} \beta_{i+1} M_i)$$

In the above equation, T is a linear time trend and $D_{m,i}$ represents dummy variables for each month of the year. Together, these variables establish a pre-pandemic temporal trend in death patterns for each ethno-racial group, while accounting for seasonal fluctations in death patterns. The r package ‘robmixglm’ was used to estimate robust regression models that account for overdispersion and minimize the influence of outlier months.

Figure C1 depicts the monthly trends in observed deaths from 2015-2021, as well as the expected deaths from the model predictions in 2020 and 2021. For all groups, a linear time trend can be observed, with the number of deaths increasing across the study period. The small number of monthly MENA deaths results in less evident cyclical patterns than with other populations. Although clear peaks of excess deaths can be observed during the primary waves of the pandemic, the month-to-month noise due to the relatively small number of deaths remains a limitation of the analysis.

Figure C1: Observed and Predicted Mortality by Ethno-Racial Group in Virginia, 2015-2022

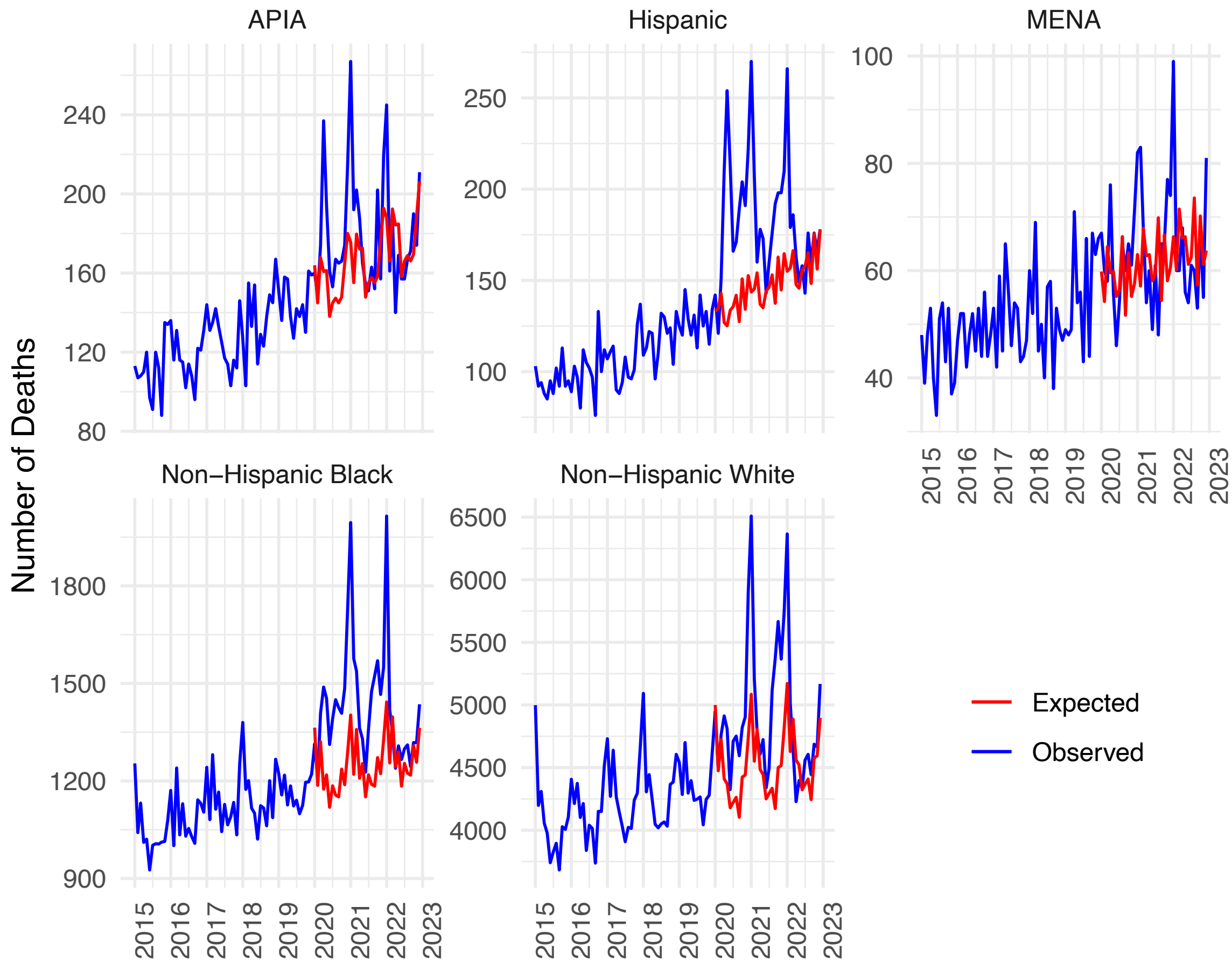

For a subset of analyses that distinguished populations by nativity, annual trends were used to calculate an expected death baseline, following the same method above. These results are reported in the main manuscript. In order to test whether there was additional heterogeneity based on the original racial classification of individuals re-classified as MENA, the model-based approach was not feasible, given the small number of deaths and the high level of month-to-month variability. Instead, a simple comparison to the 2015-2019 average number of deaths was used for comparison. These results are not reported in the main manuscript, but are presented below.

Table C1. Estimates of Excess Mortality Within the Virginia MENA Population, Disaggregated By Original Ethno-Racial Classification

			2020		2021		2022	
		2015-2019 Average Deaths	Deaths	Ratio	Deaths	Ratio	Deaths	Ratio
Foreign-Born								
	APIA	13.8	23	1.7	27	2.0	32	2.3
	Hispanic	2.0	1	0.5	1	0.5		
	Non-Hispanic Black	11.8	17	1.4	21	1.8	13	1.1
	Non-Hispanic White	279.4	360	1.3	378	1.4	257	0.9
	Other	24.6	17	0.7	34	1.4	89	3.6
U.S.-Born								
	APIA	6.6	9	1.4	6	0.9	5	0.8
	Hispanic	3.2	3	0.9	5	1.6	1	0.3
	Non-Hispanic Black	44.4	49	1.1	54	1.2	51	1.1
	Non-Hispanic White	198.8	229	1.2	230	1.2	202	1.0
	Other	2.6	4	1.5	4	1.5	6	2.3

Data come from the Virginia Department of Health vital statistics records, 2015-2022. Ratio columns represent the ratio of observed deaths in a given year to the 2015-2019 average number of deaths for each group.

The results suggest that MENA respondents previously classified as APIA had the highest rate of excess mortality, and nativity differences are present within each subpopulation. However, these results should be interpreted with caution, given the small sample sizes used for comparison. Still, given the scholarship on variation in ethno-racial

classification within the MENA population, future research may benefit from further disaggregation.

D. Virginia's MENA Population

Much of the research on the MENA population in the United States has focused on Michigan and California, two states known to have large communities of Arab and non-Arab MENA immigrants. As shown in Table D1, the MENA population of Virginia makes up 1.7% of the state's population, making it the state with the third-largest MENA population measured by relative size. In terms of absolute population numbers, Virginia's population of approximately 141,000 MENA residents would rank seventh in the United States.

Table D1. Size of MENA Population in States with the Largest MENA Population, 2019

	MENA Population	Percent of Population
Michigan	247,595	2.5
California	784,115	2.0
Virginia	141,528	1.7
New Jersey	144,107	1.6
District of Columbia	10,678	1.5
Massachusetts	100,186	1.5
New York	297,678	1.5
Illinois	136,113	1.1
Maryland	66,064	1.1
Arizona	68,916	1.0

Note: Population estimates calculated using the 2019 American Community Survey.

As Figure D1 shows, the MENA population of Virginia has been growing, and the 2020 Covid-19 pandemic does not seem to have disrupted prior population growth trends. With the exception of 2020, the MENA population appears to have grown steadily

in line with the pre-pandemic trend. Although the ACS provides experimental weights for 2020 estimates, the Census Bureau cautions against comparing 2020 estimates with other years, due to greater non-response issues during the pandemic, particularly for smaller subpopulations. While it is possible that the population fluctuated during the pandemic, the return to the trendline in 2021 and 2022, estimates suggests that the dip was related to a data collection issue, which limits the ability to calculate population denominators for mortality rates.

Figure D1. Population Estimates from the American Community Survey by Race and Ethnicity, 2015-2022

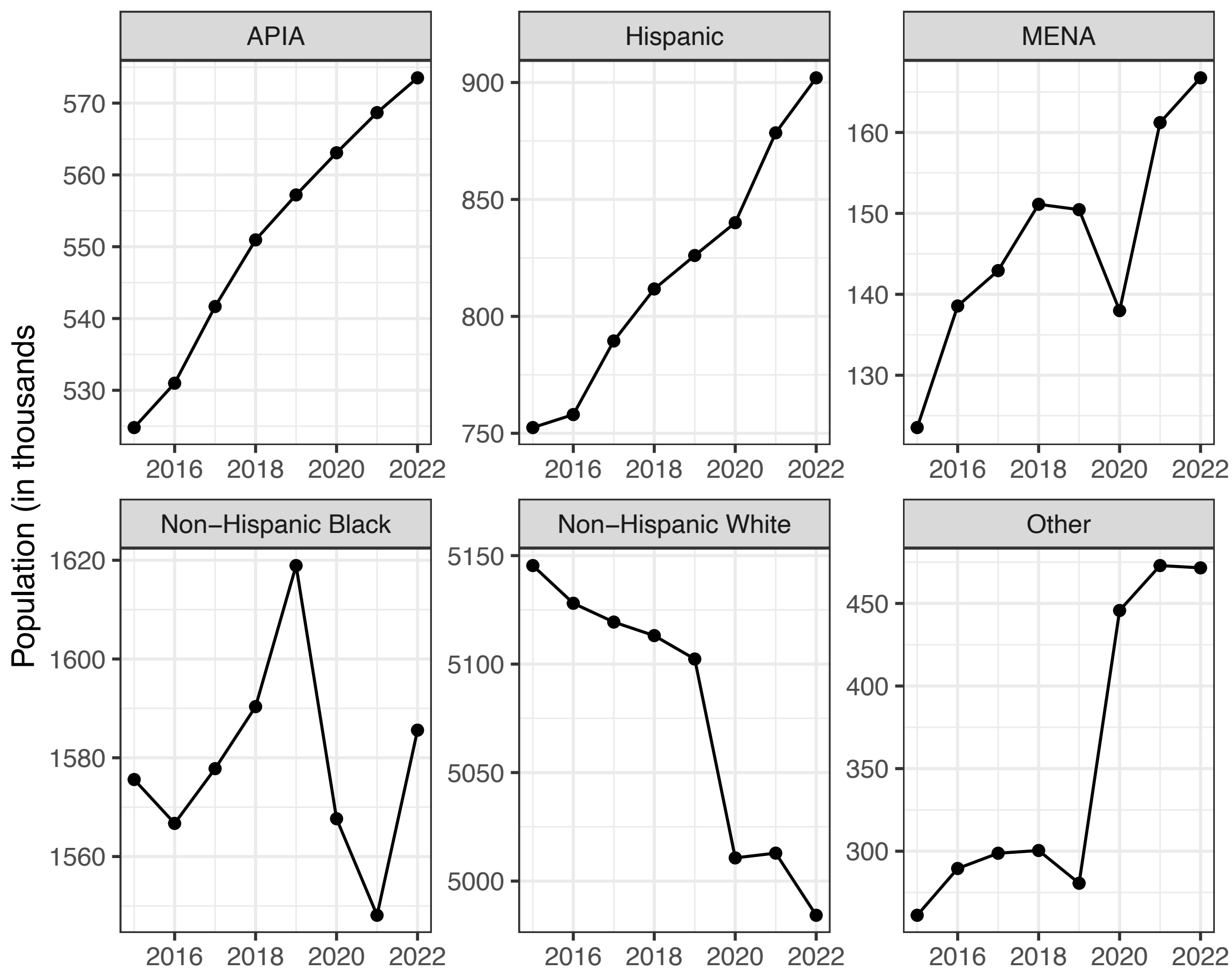

Note: Population estimates calculated using 2015-2022 American Community Survey data.

Figure D2 plots the growth rate of both deaths (observed and predicted), as well as the MENA population, relative to 2019. Because of the unreliable ACS estimates, this figure includes a smoothed population estimate based on a straightforward regression on the population data. Both the predicted number of deaths (based on a model of pre-pandemic trends) and the smoothed population estimates for 2020 were about 3-4% higher than in 2019, yet the observed number of deaths was more than 9% higher than 2019 deaths. This suggests the excess deaths of 2020-2022 were not driven by population during that period.

Figure D2. Relative Change in Population and Death Estimates, 2019-2022

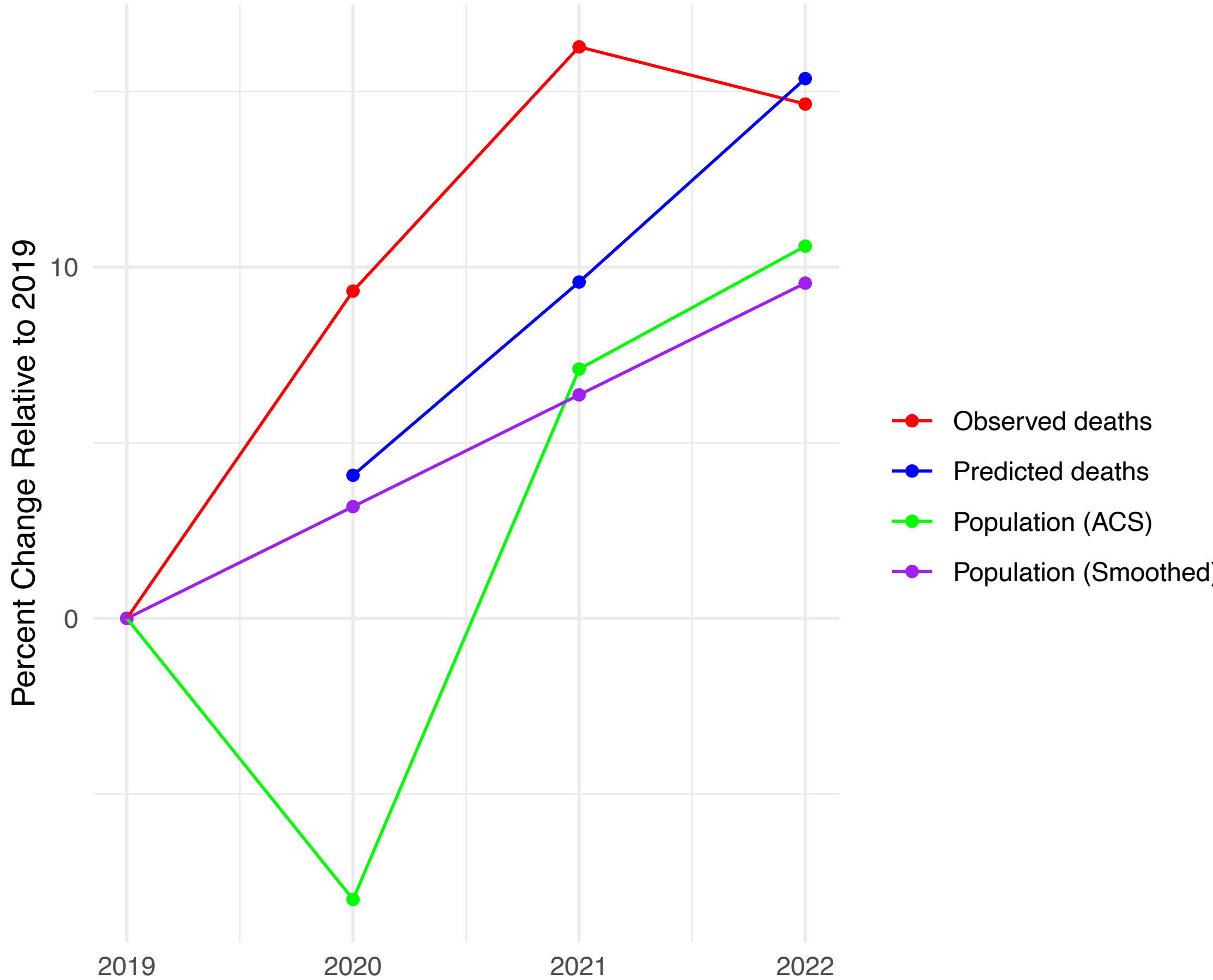

Note: Population estimates calculated using 2015-2022 American Community Survey data and included a linear smooth estimate to account for measurement discrepancies during the pandemic.

The spatial distribution of the MENA population may have also affected their experiences of the pandemic. Virginia's MENA population is highly concentrated in a few counties in northern Virginia, outside the greater Washington D.C. metro area. The map in Figure D3 shows the spatial distribution of deaths (population data is not available at the county level). In the full 2015-2022 dataset, one-third of all deaths were recorded in one county, Fairfax County, and 61% of all MENA deaths were recorded in the other counties and independent cities that makeup the Northern Virginia area. Moreover, 95%

of the MENA population lives in a metropolitan area, either in the central city or the greater metropolitan area, according to the ACS data. Approximately 84% of all MENA deaths were recorded in a county that Joe Biden won in the 2020 presidential election.

Figure D3. Distribution of MENA Deaths Across Virginia Counties

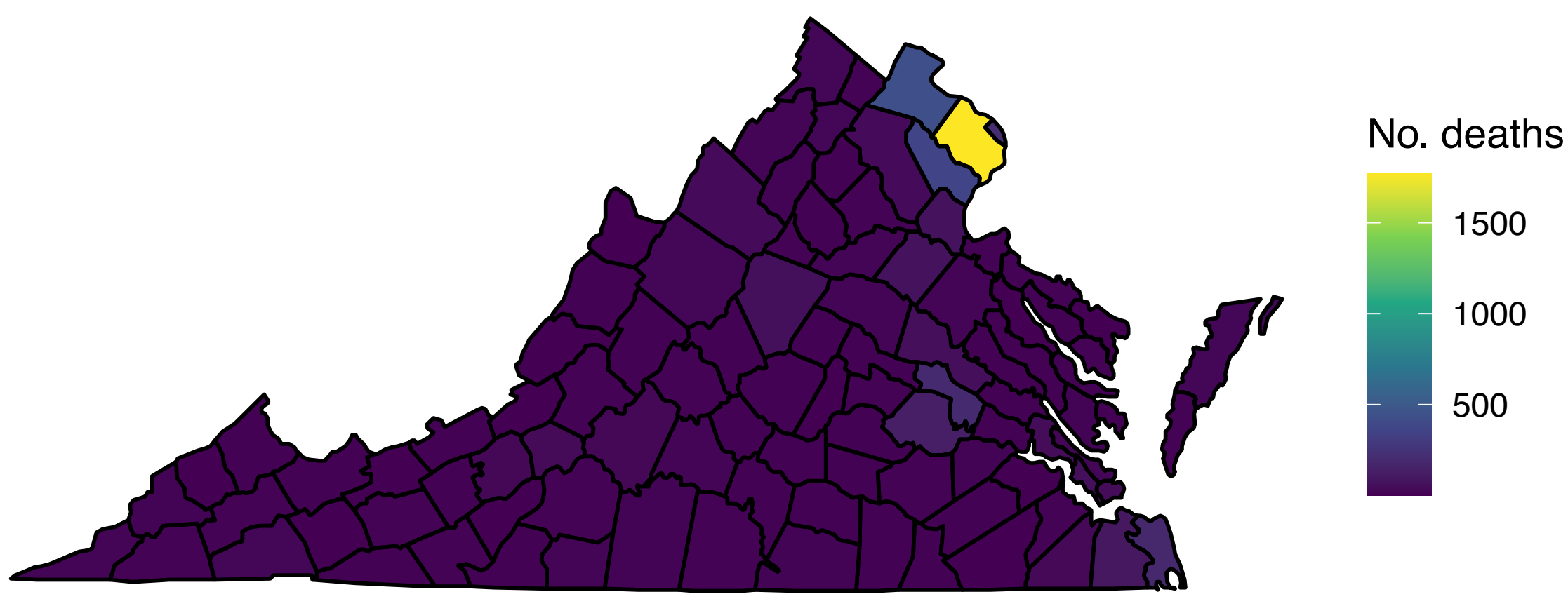

Data represent the number of deaths among the MENA population of Virginia.
